# Symmetrical (SDMA) and asymmetrical dimethylarginine (ADMA) in sepsis: high plasma levels as combined risk markers for sepsis survival

**DOI:** 10.1186/s13054-018-2090-1

**Published:** 2018-09-19

**Authors:** Martin Sebastian Winkler, Axel Nierhaus, Gilbert Rösler, Susanne Lezius, Olaf Harlandt, Edzard Schwedhelm, Rainer H. Böger, Stefan Kluge

**Affiliations:** 1 Department of Anesthesiology, University Medical Center Hamburg-Eppendorf, Martinistr. 52, 20246 Hamburg, Germany; 2 Department of Intensive Care Medicine, University Medical Center Hamburg-Eppendorf, Martinistr. 52, 20246 Hamburg, Germany; 3 Institute of Medical Biometry and Epidemiology, University Medical Center Hamburg-Eppendorf, Martinistr. 52, 20246 Hamburg, Germany; 4 Department of Internal Medicine II, Asklepios Klinik Nord-Heidberg, Tangstedter Landstr. 400, 22417 Hamburg, Germany; 5 Institute of Clinical Pharmacology and Toxicology, University Medical Center Hamburg-Eppendorf, Martinistr. 52, 20246 Hamburg, Germany; 6 Center for Anesthesiology and Intensive Care Medicine, University Medical Center Hamburg-Eppendorf, Martinistr, 52 20246 Hamburg, Germany

**Keywords:** Symmetric dimethylarginine, Asymmetric dimethylarginine, Sepsis

## Abstract

**Background:**

Nitric oxide (NO) regulates processes involved in sepsis progression, including vascular and immune function. NO is generated by nitric oxide synthases (NOS) from L-arginine. Cellular L-arginine uptake is inhibited by symmetric dimethylarginine (SDMA) and asymmetric dimethylarginine (ADMA) is a competitive inhibitor of NOS. Increased inhibitor blood concentrations lead to reduce NO bioavailability. The aim of this study was to determine whether plasma concentrations of SDMA and ADMA are markers for sepsis survival.

**Method:**

This prospective, single center study involved 120 ICU patients with sepsis. Plasma SDMA and ADMA were measured on admission (day 1), day 3 and day 7 by mass spectrometry together with other laboratory markers. The sequential organ failure assessment (SOFA) score was used to evaluate sepsis severity. Survival was documented until day 28. Groups were compared using the Mann-Whitney U test, chi-squared test or non-parametric analysis of variance (ANOVA). Mortality was assessed using Kaplan-Meier curves and compared using the log-rank test. Specific risk groups were identified using a decision tree algorithm.

**Results:**

Median plasma SDMA and ADMA levels were significantly higher in non-survivors than in survivors of sepsis: SDMA 1.14 vs. 0.82 μmol/L (*P* = 0.002) and ADMA 0.93 vs. 0.73 μmol/L (*P* = 0.016). ANOVA showed that increased plasma SDMA and ADMA concentrations were significantly associated with SOFA scores. The 28-day mortality was compared by chi-square test: for SDMA the mortality was 12% in the lower, 25% in the intermediate and 43% in the 75th percentile (*P* = 0.018); for ADMA the mortality was 18–20% in the lower and intermediate but 48% in the 75th percentile (*P* = 0.006). The highest mortality (61%) was found in patients with plasma SDMA > 1.34 together with ADMA levels > 0.97 μmol/L.

**Conclusions:**

Increased plasma concentrations of SDMA and ADMA are associated with sepsis severity. Therefore, our findings suggest reduced NO bioavailability in non-survivors of sepsis. One may use individual SDMA and ADMA levels to identify patients at risk. In view of the pathophysiological role of NO we conclude that the vascular system and immune response are most severely affected when SDMA and ADMA levels are high.

## Background

Sepsis is a life-threatening dysregulated host response to infection [1]. Disease severity may be indicated by the sepsis-related organ failure assessment (SOFA) score which defines organ dysfunction by a subset of clinical findings and laboratory markers [1, 2]. Despite improvements in sepsis treatment, such as fluid resuscitation and early antibiotics, the in-hospital mortality remains high and in Germany up to 24% of patients with sepsis die [3, 4]. The high mortality rate, however, follows variable phases of sepsis severity and physicians face a diagnostic and prognostic challenge: Patients with sepsis are difficult to identify and the course of sepsis is often unpredictable [5].

Analysis of the signaling molecule nitric oxide (NO) may provide a promising approach to the identification of high-risk cases because changes in NO levels relate to both circulatory failure and infection control [6, 7]. First, endothelial-derived NO dilates blood vessels via activation of guanylate cyclase, which induces relaxation of vascular smooth muscle cells by increasing intracellular 3,5-cyclic guanosine monophosphate (cGMP) concentration. On the one hand, excessive production of NO may cause severe hypotension with loss of systemic vascular resistance (SVR) and consequently reduced organ perfusion. On the other hand, NO is essential for maintaining microvascular function since it regulates the supply and distribution of oxygen and nutrients throughout all tissues [8]. In this context, NO maintains microvascular homeostasis by dilating and regulating vascular tone, red blood cell deformability, and leukocyte and platelet adhesion to endothelial cells [8].

Second, NO is essential to the innate immunological response to pathogens as indicated by extensive studies of innate immunity involving monocytes and macrophages [6]. NO is a free radical and has immediate antimicrobial effects including disruption of bacterial target structures and inhibition of bacterial metabolism [9–11]. NO is generated by three tissue-specific NO synthases (NOS) termed endothelial (eNOS), neural (nNOS) and inducible NOS (iNOS), the latter being expressed in immune tissue [12]. Two non-proteinogenic amino acids inhibit NO formation via interference with the NOS substrate arginine: high concentration of symmetric dimethylarginine (SDMA) inhibits cellular arginine uptake and asymmetric dimethylarginine (ADMA) competes for catalytic substrate conversion [13]. SDMA is metabolized in the kidneys by alanine-glyoxylate aminotransferase 2 (AGXT2) and is closely associated with renal function [14]. This is one reason why SDMA may serve as a reliable marker for renal failure [15, 16]. ADMA levels are controlled by two cleaving enzymes, dimethylarginase-dimethylalaminohydrolase-1 and 2 (DDAH1 and 2), of which DDAH2 is predominantly expressed in immune cells [15, 17]. Interestingly, in studies of polymicrobial sepsis in mice, global knockout of DDAH2 is associated with 12% 120-h survival compared to 53% survival in wild-type animals, underlining the important immunosuppression effect of ADMA in sepsis [18]. Further, we have shown that plasma ADMA levels are increased whereas DDAH2 expression in peripheral blood monocytes (PBMC) is reduced in patients with sepsis [19].

With this background of plasma SDMA levels depending on renal function and plasma ADMA levels depending on DDAH2 activity in immune cells, we sought to investigate whether plasma concentrations of SDMA and ADMA are associated with sepsis survival and could be useful markers to identify patients at high risk.

## Methods

### Study population

From February 2012 until December 2013, we enrolled 120 patients (> 18 years old) with sepsis admitted to the intensive care units (ICU) of the University Medical Center Hamburg-Eppendorf (Hamburg, Germany) after informed consent had been obtained from patients or their legal representatives. The local Research Ethics Committee approved the study protocol (Hamburg Chamber of Physicians, reference PV3927). The study was registered in http://www.clinicaltrials.org under the reference NCT01632059. Inclusion criteria were diagnosed infection, a clinical syndrome pathognomonic for an infection or the suspicion of infection. The available sepsis criteria from the American College of Chest Physicians/Society of Critical Care Medicine were originally used to identify patients with sepsis [20]. Following publication of the consensus Sepsis-3 guidelines (which were not available during the study) we generated sepsis-related organ failure assessment (SOFA) scores for all patients to meet the latest sepsis criteria. Exclusion criteria were age < 18 years, other forms of shock, pregnancy or moribund disease status.

### Clinical evaluations and assays

SOFA scores were calculated on admission (day 1) and on day 3 and day 7, if patients stayed at least 3 days in the ICU. Within the first 24 h after inclusion, basic demographic and clinical data were recorded. Blood was drawn to measure plasma SDMA and ADMA and to determine other clinical parameters including leukocyte counts, creatinine (crea), lactate, C-reactive protein (CRP), procalcitonin (PCT) and interleukin-6 (IL-6). All routine clinical assays were performed at the Department of Clinical Chemistry at the University Hospital Hamburg-Eppendorf (Hamburg, Germany). All blood samples were processed identically and plasma SDMA and ADMA concentrations were determined by liquid chromatography (LC)-tandem mass spectrometry (MS) analysis as described previously [21, 22]. Briefly, 25 μL aliquots of plasma were spiked with stable isotope-labeled ADMA, which served as the internal standard. Proteins were precipitated with 100 μL of methanol, filtered through a 0.22 μm hydrophilic membrane (Multiscreen HTS™, Millipore, Molsheim, France), derivatized with butanolic 1 N HCl, and analyzed by LC-tandem MS (Varian 1200 MS, Agilent Technologies, Santa Clara, USA). Quantification was performed by calculation of peak area ratios and calibration with known concentrations of analytes in dialyzed EDTA plasma. The analytical range of the method was validated for 0.05–4 μmol/L and the coefficient of variation was ≤ 7.5% both for ADMA and for SDMA [21, 22].

### Statistical analysis

The primary variables were SDMA and ADMA concentrations in plasma (expressed in micromole per liter). These and other continuous variables are reported as median and interquartile range (IQR). Differences between groups were tested for significance using either the Mann-Whitney U test for two groups or the Kruskal-Wallis test (non-parametric analysis of variance (ANOVA)) for more than two groups and trend analysis. Spearman’s rank correlation was used to assess pairwise correlations. Survival analyses were performed comparing three groups: (I) lower (≤ 25th percentile), (II) intermediate (> 25–75th percentile) and (III) upper quartiles (> 75th percentile) of baseline plasma concentrations both of SDMA and of ADMA. Mortality was assessed using Kaplan-Meier curves and 28-day-survival was compared using the chi-squared test. We further have used the quick, unbiased, efficient statistical tree (QUEST) method, which is a tree-building method for nominal dependent variables that avoids bias inherent in other methods in favor of predictors with many categories. It is based on the chi-square test for categorical or the *F* test for continuous variables, and produces binary node splits. Here, the significance level for node splitting was set to 0.05 and parent groups had a minimum size of 10 patients while child nodes had to have at least 5 patients. Included independent variables were day-1 values of SDMA in quartiles and ADMA in quartiles. The method has been described by Loh and Shih and recently reviewed by other researchers [23, 24]. A detailed method description is also available in the IBM manual for SPSS (2013): *IBM SPSS Statistics for Windows, Version 22.0. Armonk, NY: IBM Corp., Manual IBM SPSS Decision Trees 24.* For all analyses, a *P* value <0.05 was considered to be statistically significant and all calculations were performed using SPSS version 24 (IBM SPSS Inc. Cary, NC, USA) and Graph Pad version 7 for Mac (GraphPad Software, La Jolla CA, USA).

## Results

Plasma SDMA and ADMA concentrations were measured in 120 patients diagnosed with sepsis and admitted to the ICU. During follow up, plasma samples from 106 patients at day 3 and from 71 patients at day 7 were obtained before discharge or death (Table 1). A total of 31 patients (26%) died within 28 days; for further analysis, the cohort was divided into 28-day survivors and non-survivors. There was no difference between groups in basic demographic data, age and gender, cause of sepsis (medical or surgical) or the species detected or gram positivity or negativity (Table 1). However, non-survivors had a higher SOFA score than survivors (Table 1, *P* = 0.006).

**Table 1:** Baseline characteristics of the study population

Characteristic	All patients	Survivors 28 days	Non-survivors 28 days	*P* value^b^
Admission day 1, *n* (%)	120 (100)	89 (74)	31 (26)	N/A
ICU day 3, *n* (% from day 1)	106 (88)	78 (88)	28 (90)	N/A
ICU day 7, *n* (% from day 1)	71 (59)	56 (63)	15 (50)	N/A
Age, years^a^	63 (53–74)	63 (53–74)	68 (51–75)	0.392^b^
Male/female, *n* (%)	86/34 (72/38)	62/17 (70/30)	24/7 (77/23)	0.903
Medical/surgical admission, *n* (%)	64/56 (53/67)	43/46 (48/52)	21/10 (68/32)	0.062
Gram+/gram- bacteria, *n* (%) (*n* = 94)	46/36 (49/38)	37/25 (53/36)	9/11 (38/46)	0.250^c^
SOFA score^a^	10 (7–14)	9 (6–13)	12 (8–17)	0.006

*ICU* Intensive Care Unit, *SOFA* sepsis related organ failure assessment score, *N/A* not applicable

^a^Data are presented as median (interquartile range, IQR)

^b^Non-parametric Mann-Whitney U test comparing 28-day survivors with non-survivors

^c^Chi-squared test comparing 28-day survivors with non-survivors

### Plasma SDMA and ADMA levels were higher in non-survivors

**Fig. 1:**
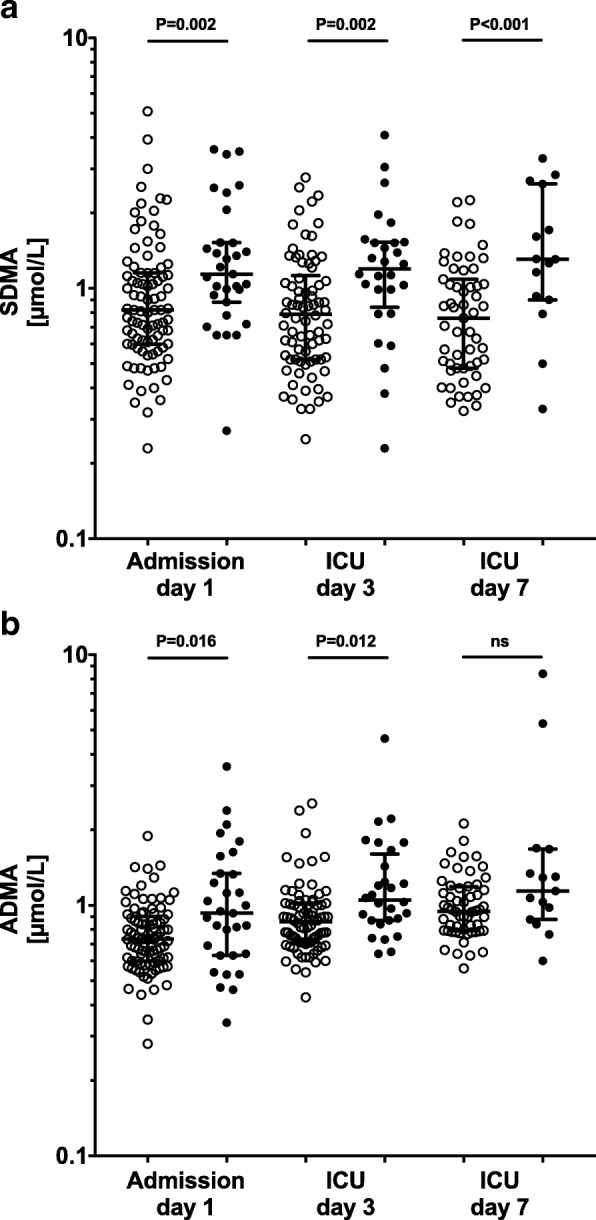
Plasma concentrations of symmetric (SDMA) and asymmetric dimethylarginine (ADMA) in sepsis survivors and non-survivors. **a** SDMA and (**b**) ADMA levels in 28-day survivors and non-survivors were compared on admission (day 1), day 3 and day 7. **a** SDMA levels were significantly higher in non-survivors than in survivors on all days. **b** ADMA levels were significantly higher on admission and on day 3 in non-survivors. The 28-day survivors (circles) and non-survivors (dots) were compared using the non-parametric Mann-Whitney U test. Plots show median and interquartile range (IQR). ns, non-significant

We measured plasma concentrations of SDMA and ADMA together with various other inflammatory and sepsis severity markers. Median plasma SDMA and plasma ADMA concentrations were significantly increased in non-survivors at day 1, 3 and 7 (Fig. 1): on admission (day 1) the median plasma concentration of SDMA was increased by 40% (*P* = 0.002) and of ADMA by 27% (*P* = 0.016) in non-survivors. Table 2 provides an overview of all variables for day 1: procalcitonin (PCT), CRP and IL-6 were significantly higher in non-survivors (Table 2). The laboratory markers included in the SOFA score (creatinine and bilirubin) were significantly higher (Table 2; *P* < 0.01) but platelet counts were significantly lower in non-survivors (Table 2; *P* < 0.01). Variables were analyzed for their association with plasma SDMA and ADMA using Spearman’s rank correlation test (Table 3). Plasma SDMA and plasma ADMA concentrations correlated positively with creatinine, bilirubin and lactate. In addition, plasma SDMA correlated positively with PCT and negatively with the platelet count.

**Table 2:** Laboratory parameters on admission to ICU, day 1

Parameter	All patients	Survivors 28 days	Non-survivors 28 days	*P* value^a^
SDMA, μmol/L	0.93 (0.65–1.34)	0.82 (0.60–1.15)	1.14 (0.88–1.52)	0.002*
ADMA, μmol/L	0.77 (0.60–0.97)	0.73 (0.60–0.90)	0.93 (0.63–1.34)	0.016*
Leukocytes, ×10^9^/L	12.6 (7.5–18.9)	12.8 (9.4–19.2)	12.3 (6.2–18.2)	0.205
C-reactive protein, mg/L	186.5 (124.0–243.8)	182.0 (97.5–248.0)	195.0 (130.0–243.0)	0.516
Procalcitonin, μg/L	2.25 (0.70–8.25)	1.49 (0.51–6.66)	6.29 (1.19–19.64)	0.003*
Interleukin-6, ng/L	241 (79–614)	216 (61–554)	278 (111–1000)	0.075
Lactate, mmol/L	1.1 (0.8–1.8)	1.0 (0.8–1.5)	1.2 (0.9–2.4)	0.081
Creatinine, mg/dL	1.2 (0.8–2.3)	0.6 (0.7–1.9)	1.7 (1.1–2.6)	0.009*
Bilirubin, mg/dL	0.7 (0.3–1.5)	0.6 (0.3–1.0)	1.4 (0.6–3.2)	< 0.001*
Platelets, ×10^9^/mL	183 (93–280)	199 (124–290)	107 (40–274)	0.005*

Data are presented as median (interquartile range, IQR)

*SDMA* symmetric dimethylarginine, *ADMA* asymmetric dimethylarginine

^a^Non-parametric Mann-Whitney U test comparing 28-day survivors with non-survivors

*Statistically significant

**Table 3:** Spearman’s rank correlation between SDMA, ADMA and various laboratory parameters

Parameter	SDMA rho (95% CI)	*P* value	ADMA rho (95% CI)	*P* value
Leucocytes	0.05 (− 0.14 to 0.23)	0.612	0.07 (− 0.12 to 0.25)	0.447
C-reactive protein	0.12 (− 0.07 to 0.29)	0.208	−0.08 (− 0.26 to 0.11)	0.405
Procalcitonin	0.47 (0.31 to 0.60)	<0.0001*	0.08 (−0.11 to 0.26)	0.415
Interleukin-6	0.09 (−0.10 to 0.27)	0.349	0.02 (−0.17 to 0.20)	0.844
Lactate	0.24 (0.05 to 0.40)	0.0066*	0.23 (0.05 to 0.40)	0.007*
Creatinine	0.72 (0.62 to 0.80)	<0.0001*	0.20 (0.02 to 0.37)	0.289
Bilirubin	0.26 (0.08 to 0.42)	0.0041*	0.19 (0.003 to 0.36)	0.039*
Platelets	−0.20 (−0.37 to −0.017)	0.027*	−0.17 (− 0.34 to 0.02)	0.072

Spearman’s rho correlation coefficient is presented with 95% confidence interval (95% CI)

(*statistically significant)

*SDMA* symmetric dimethylarginine, *ADMA* asymmetric dimethylarginine

### Plasma SDMA and ADMA concentrations are associated with severity of sepsis

**Fig. 2:**
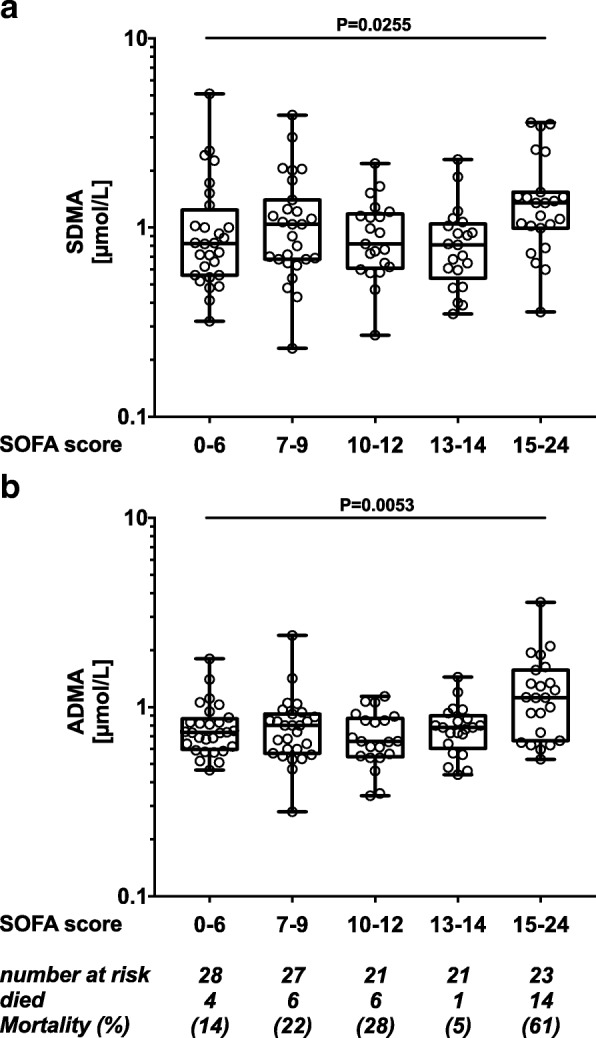
Plasma symmetric dimethylarginine (SDMA) and asymmetric dimethylarginine (ADMA) in different sepsis severity groups. Patients were grouped according to their individual sequential organ failure assessment (SOFA) score and SDMA concentrations (**a**) and ADMA concentrations (**b**) are shown for day 1. Both SDMA and ADMA levels showed a trend towards increasing levels in patients with higher SOFA scores. Groups were compared using the Kruskal-Wallis test for trend analysis. Plots show median and interquartile range (IQR)

The comprehensive SOFA score is the gold standard for evaluating sepsis severity. We used the SOFA score to create five sub-groups reflecting increasing disease severity. ANOVA showed a significant association between plasma SDMA and ADMA levels and SOFA scores (Fig. 2). The highest median SDMA and ADMA concentrations occurred in severely sick patients with SOFA scores > 15. Accordingly, in this group of 23 patients, 14 (61%) did not survive sepsis.

### Low levels of plasma SDMA and ADMA are predictive of sepsis survival

**Fig. 3:**
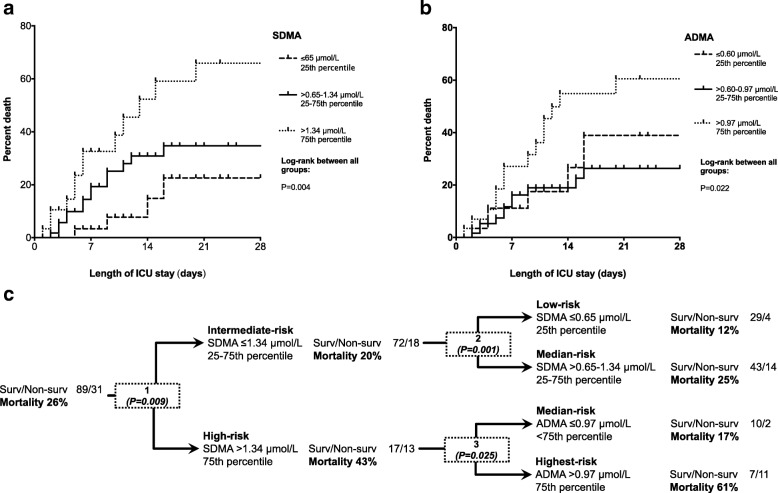
Survival of patients with sepsis in quartile groups of plasma symmetric (SDMA) and asymmetric dimethylarginine (ADMA) concentration. Mortality curves were calculated for 28-day survival and three groups were compared: patients with SDMA levels (**a**) or ADMA levels (**b**) ≤ 25th percentile, between 25th and the 75th percentile (interquartile) and > 75th percentile. **a** The three groups differed significantly (*P* = 0.004): patients with SDMA levels > 1.34 μmol/L had the highest mortality and patients with SDMA levels ≤ 0.65 μmol/L the lowest mortality. **b** There was no difference in mortality between patients in the interquartile and ≤ 25th percentile (*P* = 0.715); however, patients with ADMA levels > 75th percentile had the highest mortality compared to other groups (*P* = 0.022). **c** Decision tree to identify patient risk. Out of 120 patients, 31 died (26%). First decision knot: (1) patients were identified as having intermediate risk, when SDMA levels were ≤ 1.34 μmol/L. This group was further risk stratified by SDMA levels; (2) low-risk patients had levels ≤ 0.65 and median-risk patients had levels between 0.65 and 1.34 μmol/L. Mortality increased to 43% when SDMA levels were > 1.34 μmol/L, indicating high risk. At this step ADMA levels may help to identify patients with the highest risk of not surviving sepsis; (3) plasma ADMA concentrations > 0.97 μmol/L were associated with a 61% mortality rate

We plotted mortality curves to demonstrate cumulative survival by subgroup. We divided the cohort according to plasma SDMA and ADMA concentrations into three groups: plasma levels ≤ 25th percentile, between the 25 and 75th percentile (intermediate) and > 75th percentile. Figure 3 shows Kaplan-Meier mortality curves for SDMA (Fig. 3a) and ADMA (Fig. 3b) in those three groups. Most patients died within 14 days after ICU admission. Survival was significantly different (*P* = 0.004) between sub-groups based on SDMA concentrations (Fig. 3a). Within 28 days 12% of patients with plasma SDMA concentrations ≤ 0.65 μmol/L died, but 43% of those with plasma SDMA levels > 1.34 μmol/L died (Fig. 3a); 25% of patients between those extremes (25–75th percentile) died. There was no significant difference in mortality rate in the low (20%) and interquartile group (18%, *P* = 0.715) when ADMA levels were analyzed (Fig. 3b). However, the mortality rate in the upper 75% quartile was 48% (*P* = 0.004).

Specific risk groups were identified using a decision tree model (QUEST) taking plasma SDMA and ADMA concentrations as markers to identify patients at risk. We combined the findings presented in Kaplan-Meier curves in a decision tree to classify (I) low, (II) intermediate and (III) high-risk patients on ICU admission (Fig. 3c). In a first step, knowledge of SDMA level alone can help to define risk groups, as patients with SDMA ≤ 1.34 μmol/L are considered as having intermediate risk: 18 out of 90 patients (20%) in this category died. In the intermediate-risk group we identified low-risk patients when SDMA levels were ≤ 0.65 μmol/L and median-risk patients when SDMA levels were between 0.65 and 1.34 μmol/L, with 12% and 25% mortality. Patients with the highest SDMA concentrations > 1.34 μmol/L are at high risk and mortality in these patients reached 43%. However, in these patients ADMA concentrations may help to identify those with the very highest risk. When plasma SDMA concentrations were > 1.34 μmol/L together with ADMA concentrations > 0.97 μmol/L, mortality was 61%, whereas with ADMA concentrations ≤ 0.97 μmol/L the chances of surviving sepsis were median.

## Discussion

The aim of this study was to determine whether plasma SDMA and plasma ADMA concentrations are associated with sepsis survival and are useful markers to stratify risk levels. We found that elevated levels of SDMA and ADMA in non-survivors were associated with disease severity. The occurrence of high plasma SDMA together with high plasma ADMA levels identified individuals at risk of a poor outcome who might need the highest priority of care.

The most important factors affecting sepsis outcome are circulatory failure and a disturbed host response [1]. NO is an important signaling molecule with regulatory functions in both the circulatory and immune systems. We were therefore interested to determine whether plasma concentrations of SDMA and ADMA, which are direct and indirect inhibitors of NOS, are altered in sepsis and hypothesized that increased levels might contribute to lower NO bioavailability.

Physiologically, NO regulates blood pressure and is the most important vasodilatator. Reduced NO levels are responsible for progression of a variety of conditions associated with hypertension such as coronary heart disease and chronic renal failure. Reduced NO levels have been attributed to an increase in SDMA and ADMA concentrations, which are established risk markers for cardiovascular mortality [25, 26]. Furthermore, ADMA infusions in humans result in increased systemic vascular resistance (SVR) and mean arterial pressure (MAP) [27]. In contrast, one hallmark of sepsis and septic shock is loss of SVR and MAP with subsequent alterations in cardiac output (CO) and reduced tissue perfusion [28]. This leads to the hypothesis that, in sepsis, NO production is unregulated and may be responsible for vascular failure [29, 30]. We have previously shown that NO bioavailability is reduced in sepsis; this is indicated by low NOS substrate levels but high levels of the NOS inhibitor ADMA; we are now able to provide data for SDMA [19]. The theory that low NO levels pertain to sepsis severity is supported by a randomized clinical trial in which a NOS inhibitor was tested to stabilize hemodynamic derangements in septic patients. Intravenous administration of the non-selective NOS inhibitor L-NG-methyl-L-arginine hydrochloride (546C88) has been compared with placebo. The 546C88 increased SVR but other hemodynamic parameters such as CO and oxygen delivery were blunted. In the long term, administration of 546C88 was associated with higher sepsis mortality and despite macrocirculatory improvements during treatment, the drug was not associated with favorable sepsis outcomes [31, 32].

How can we explain the apparently contradictory roles of NOS? Inhibition of NOS in the macrocirculation may improve signs of hypotension and loss of SVR but inhibition in the microcirculation may impair capillary exchange resulting in organ dysfunction [12]. Thus, the effect of NO is ambivalent in sepsis. However, the protective effect of NO on the microvascular exchange seems to predominate [33–37].

SDMA is transported efficiently by the hCAT-2B transporter into cells and exchanged against intracellular L-arginine, resulting in cellular L-arginine depletion in vitro [38]. Although we cannot exclude the possibility that SDMA concentrations, e.g. in the microcirculation, are higher than plasma concentration and may influence NO synthesis locally; this depletion mechanism requires very high SDMA concentrations, i.e. 5 mM, much higher than those observed in our study [38]. Moreover SDMA, may amplify its diagnostic role as a marker for organ function as elimination depends entirely on renal function, which is extremely sensitive to the septic assault. Acute kidney injury (AKI) occurs in over 40% of patients [39, 40]. Considering this important effect, one may argue that early detection of septic AKI is of great importance. The SOFA score includes a variety of clinical parameters among which creatinine (Crea) reflects renal function [41]. However, serum Crea as a renal marker in sepsis is questionable because of the well-known problem that serum Crea levels are influenced by various factors that are altered in critical illness and supported by experimental data in mice showing that Crea levels are reduced after induction of sepsis, which may mask early detection of AKI [42, 43]. However, SDMA is a very established and superior marker for kidney function [44]. In a meta-analysis involving over 2100 patients, the coefficient of correlation between SDMA and inulin clearance (the gold standard for measuring kidney function) was 0.85 (95% CI 0.76–0.91) [16]. In this context, one may consider that SDMA is not only a molecule cleared by the kidneys but that SDMA accumulation reflects the degree of renal failure, and high concentrations potentially may inhibit NOS to a greater extent [34]. In sepsis, this relationship may reflect sustained and disturbed microcirculatory exchange with consequent impairment of organ function. We have observed those associations and shown that SDMA concentrations correlate not only with Crea but also with liver failure (bilirubin), the collapse of coagulation as represented by platelet counts and the SOFA score (Table 3, Fig. 2). Moreover, our results are supported by those of two previous studies involving 223 and 67 patients with sepsis, respectively, in which SDMA levels were elevated and associated with higher mortality [45, 46]. The positive correlation between SDMA and PCT might be explained by the fact that PCT elimination is prolonged in patients with renal dysfunction [47]. Creating risk groups on the basis of SDMA levels seems an appropriate tool to identify patients needing early and high priority of care such as hemodialysis or intensified hemodynamic monitoring (Fig. 3).

Beside the important role of NO levels in circulation, reduced levels of NO might be deleterious for the immune response in sepsis and increased NOS inhibitor levels may contribute to this. It has long been observed that iNOS knockout (removing the predominant NOS in immune tissue) is associated with poor survival in mice with sepsis [48]. This effect is reversed by transplantation of wild-type bone marrow to iNOS knockout mice [49]. Increased release of cytokines such as TNF-α together with better survival was observed in mice with pneumonia when iNOS was restored this way [49]. Together with the discovery that mouse macrophages produce large amounts of nitrite (NO2-) and nitrate (NO3-) upon bacterial lipopolysaccharide (LPS) stimulation, it has been suggested that decreased NO levels impair the innate immune response under septic conditions [50]. The reduced ability of monocytes to release pro-inflammatory cytokines after stimulation with LPS indicates the immunosuppressive phase of sepsis in humans and has been suggested as a standard tool to identify immunosuppressed patients [51, 52]. In summary, it seems that manipulation of NO metabolism either by knocking out iNOS or by high levels of NOS inhibitors such as ADMA is associated with sepsis severity and mortality [19]. ADMA levels were associated with mortality in two studies of 267 patients with sepsis and 255 critically ill patients, and in a small population of patients with sepsis the odds ratio for 28-day mortality in the upper quartile was 20.8 [45, 53, 54].

While SDMA levels depend on renal function, ADMA clearance is directly linked to the immune system and levels are controlled by DDAH1 and DDAH12 activity, of which DDAH2 is predominantly expressed in immune cells [15, 17]. Circulating ADMA levels are genetically determined by promoter polymorphism in a regulatory gene encoding DDAH2 polymorphism and have been investigated by other research groups. In a series of 47 patients with severe sepsis, high ADMA concentration was associated with the DDAH2 -449G polymorphism [55]. Interestingly, we have previously shown an association between decreasing DDAH2 expression in peripheral blood monocytes (PBMC) and disease severity [19]. This is in line with animal experiments in DDAH2 knockout mice in which the 120-h survival rate was only 12% compared to 53% in wild-type animals after cecal ligation and puncture [18]. The authors attributed this phenotype to impaired macrophage function as monocyte-specific deletion of DDAH2 results in a similar pattern of increased severity to that seen in animals globally deficient in DDAH2. The DDAH2 knockout in macrophages was associated with a significantly higher bacterial load in plasma and in the peritoneum. NO production in activated mouse macrophages is DDAH2-dependent with reduced intracellular NO levels within the cell, which impairs motility and phagocytosis in these cells [18].

Increased SDMA and ADMA levels directly correlate with sepsis severity and mortality. We have used this information to suggest a risk classification (Fig. 1 and Fig. 3). Among our patients, 17% died when SDMA levels were ≥ 1.34 and ADMA levels were ≤ 0.97; the highest mortality however (67%) was observed when ADMA levels were > 0.98 μmol/L (Fig. 3). One may hypothesize that NOS inhibitors reflect two essential pathophysiological steps in sepsis: vascular failure and immunosuppression. Thinking about sepsis markers it is important to combine markers for both vascular function and for immunity. Analyzing the NO metabolism is a promising approach in this context.

This was a prospective observational study and therefore had certain limitations. The study was carried out at a single center and involved relatively small numbers of patients. Future studies involving larger cohorts should eliminate potential selection bias and further statistical adjustments such as for estimated glomerular filtration rate (eGFR) should be made possible. We observed that the two direct and indirect endogenous NOS inhibitors SDMA and ADMA were associated with mortality and disease severity, which may indicate disturbances in NO metabolism. The gold standard for analysis of NO metabolism would be its direct measurement in blood. This currently seems impossible, in particular in an ICU setting, due to the short half-life of the NO molecule. Therefore, we can only speculate that ADMA and SDMA may influence NO metabolism in septic patients. Experimental studies are required, to explain why SDMA and ADMA are increased in sepsis and how this may influence NO metabolism especially in the microcirculation. Another limitation is that we cannot exclude the possibility that dialysis may influence SDMA and ADMA levels. However, we observed high SDMA and ADMA concentration in non-survivors, which warrants follow-up studies addressing the question, e.g. as to whether patients would benefit from early SDMA-guided renal replacement therapy. To date it has not been shown that dialysis improves sepsis outcome [56, 57]. Furthermore, it would be interesting to know if increased ADMA levels are associated with other acquired changes in sepsis-induced immunosuppression such as downregulation of monocytic HLA-DR receptors. Nevertheless, we believe that our observations warrant follow-up studies with larger patient groups to confirm the power of SDMA and ADMA to indicate sepsis outcome and severity.

## Conclusion

In patients with sepsis, the concentrations of SDMA and ADMA are increased. We suggest a risk classification according to SDMA and ADMA levels. Due to the biological elimination process of SDMA and ADMA one may speculate that SDMA accumulates with severity of renal failure and ADMA with severity of immune dysfunction and one may consider SDMA and ADMA as markers to indicate vascular failure and immune dysregulation in sepsis, respectively.

## Abbreviations

ADMA: Asymmetrical dimethylarginine
AGXT2: Alanine-glyoxylate aminotransferase 2
AKI: Acute kidney injury
ANOVA: One-way analysis of variance
CLP: Cecal ligation and puncture
CO: Cardiac output
Crea: Creatinine
CRP: C-reactive protein
DDAH1 and 2: Dimethylarginase-dimethylalaminohydrolase-1 and 2
eNOS: Endothelial nitric oxide synthases
ICU: Intensive Care Unit
IL-6: Interleukin-6
iNOS: Inducible nitric oxide synthases
LPS: Lipopolysaccharide
MAP: Mean arterial pressure
nNOS: Neural nitric oxide synthases
NO: Nitric oxide
NOS: Nitric oxide synthases
PBMC: Peripheral blood monocytes
PCT: Procalcitonin
QUEST: Quick, unbiased, efficient statistical tree
SDMA: Symmetrical dimethylarginine
SOFA: Sepsis-related organ failure assessment
SVR: Systemic vascular resistance
